# On Hops and Lupulin

**Published:** 1861-03

**Authors:** Charles A. Tufts

**Affiliations:** Dover, New Hampshire


*On Hops and Lupulin.* By Charles A. Tufts, of Dover, New Hampshire.

"It has been asserted that hops that have been used in obtaining the lupulin of commerce are afterwards sold as hops. Is the assertion true, and if so, to what extent is it carried on, and where; and what are the means of detecting the fraud?"

I have not been able to obtain such definite information as I could wish, in regard to this subject, and I do not think it would be easy to do so. So far as I can ascertain, it is not the practice with the hop growers and sellers in New England, and I cannot find with certainty that it is practised elsewhere.

I find there is an impression that it may be done, but I cannot learn where it is done, or by whom the fraud is committed. I have conversed with different hop-growers and dealers, and they have disclaimed all knowledge personally of such practice.

Hops, after being picked, are kiln-dried on frames, generally now by steam heat. The green hops are placed about a foot in thickness on the frames, and are often stirred to make them dry evenly. The lupulin was formerly wasted, but now most curers of hops suspend cloth under the frames, and save the lupulin which falls through.

In drying a bale of two hundred pounds weight, from one to two pounds of lupulin can be collected; to obtain more than this, it would be necessary to pass the hops a second time through the kiln. In this way four to five pounds more could be obtained. The hops would be greatly injured by this process, not only by being deprived of the lupulin, or, as hop-growers term it, "the condition," but they would be very brittle, and would be so broken as to be unsaleable. One hop-grower told me, he did not believe this was practised, as he thought the amount of lupulin would not compensate for the labor and expense of this second drying.

There is a difference in hops raised by the same grower, for this reason. As the hops are dried, they are placed in a pile in the store or curing room, till all the crop has been dried; they remain here several weeks, as the curers say to toughen; they are then pressed and bagged.

The hops at the bottom of the heap will thus have much more lupulin than those at the top, and be stronger and more valuable; considerable lupulin will be collected from the floor of the curing room, and lupulin thus collected will be of the best quality.

I have heard that quantities of lupulin are separated specially for the supply of commerce, by threshing the hops after they are a year old, and then sifting the powder from the broken strobiles.

The hops thus treated are said to be put up in bags, and then sent to auction, and sold for what they will bring, without any explanation as to their inferiority. It may be these threshed hops are again damped and pressed into pound or half pound papers.

The best way of detecting the fraud would be to remove the contents of a package or bale, in small quantities; if there was little or no lupulin left, and the strobiles were much broken, it would show they had been exposed to the above treatment.

To determine the quality of good hops, not only the color should be examined, but the powder should be rubbed between the fingers; if the lupulin is abundant and feels clammy and unctuous, and is not too dark colored, the hops may be pronounced of good quality.—*Proc. American Pharmaceutical Association*, 1860.

